# A Unified GAN-Based Framework for Unsupervised Video Anomaly Detection Using Optical Flow and RGB Cues

**DOI:** 10.3390/s25185869

**Published:** 2025-09-19

**Authors:** Seung-Hun Kang, Hyun-Soo Kang

**Affiliations:** Department of Information and Communication Engineering, School of Electrical and Computer Engineering, Chungbuk National University, Cheongju-si 28644, Republic of Korea; kksh4693@chungbuk.ac.kr

**Keywords:** deep learning, video anomaly detection, unsupervised learning, GAN, optical flow

## Abstract

Video anomaly detection in unconstrained environments remains a fundamental challenge due to the scarcity of labeled anomalous data and the diversity of real-world scenarios. To address this, we propose a novel unsupervised framework that integrates RGB appearance and optical flow motion via a unified GAN-based architecture. The generator features a dual encoder and a GRU–attention temporal bottleneck, while the discriminator employs ConvLSTM layers and residual-enhanced MLPs to evaluate temporal coherence. To improve training stability and reconstruction quality, we introduce DASLoss—a composite loss that incorporates pixel, perceptual, temporal, and feature consistency terms. Experiments were conducted on three benchmark datasets. On XD-Violence, our model achieves an Average Precision (AP) of 80.5%, outperforming other unsupervised methods such as MGAFlow and Flashback. On Hockey Fight, it achieves an AUC of 0.92 and an F1-score of 0.85, demonstrating strong performance in detecting short-duration violent events. On UCSD Ped2, our model attains an AUC of 0.96, matching several state-of-the-art models despite using no supervision. These results confirm the effectiveness and generalizability of our approach in diverse anomaly detection settings.

## 1. Introduction

Video anomaly detection (VAD) plays a critical role in surveillance and safety applications. In this study, we define an anomaly as any spatiotemporal pattern that significantly deviates from the distribution of normal training data. Typical examples include violent actions (e.g., fighting), accidents (e.g., falling), or irregular crowd behaviors (e.g., sudden dispersal). Normal events, in contrast, are routine and predictable activities observed in the training set. This definition highlights that anomalies are not predefined classes but unforeseen deviations, which makes them inherently difficult to anticipate and model.

Collecting comprehensive annotations for all possible anomalies is practically infeasible due to diversity and rarity. This has motivated unsupervised video anomaly detection (UVAD), which learns only from normal data and flags deviations at test time. Readers can find broader context and taxonomies in recent surveys on (video) anomaly detection that summarize problem settings, datasets, and evaluation protocols [1,2,3,4,5].

Traditional UVAD methods often reconstruct frames or predict future frames, using large errors as anomaly cues. However, purely reconstruction- or prediction-based models can struggle to jointly capture spatial appearance and temporal motion, limiting generalization in complex scenes. They may also overfit frequent backgrounds and inadvertently reconstruct abnormal regions with high fidelity. These challenges are documented in prior reviews and benchmarks that emphasize motion/appearance fusion and temporal reasoning as open issues [1,2].

To address these limitations, we propose a unified GAN-based framework that integrates optical flow—computed by UniMatch—and RGB appearance via a dual-encoder generator with a lightweight GRU–attention temporal bottleneck. A ConvLSTM-based discriminator evaluates temporal coherence, and a composite loss (DASLoss) combines pixel, perceptual, temporal, and feature matching terms for stable training. Compared with prior reconstruction/prediction-based baselines, our model explicitly fuses motion and appearance, enabling improved anomaly localization.

We evaluate on three benchmarks—XD-Violence, Hockey Fight, and UCSD Ped2—covering diverse scenes from crowd surveillance to sports violence. Our model attains an AP of 80.5 on XD-Violence, an AUC of 0.92 on Hockey Fight, and an AUC of 0.96 on Ped2, demonstrating cross-domain generalization under a fully unsupervised setting. For reproducibility, the source code is publicly available (see Results, Section 4).

Finally, we acknowledge an information-theoretic limitation: when the entropy of anomalous perturbations is comparable to or below the aggregate entropy of background variability and sensor noise, the mutual information between anomalies and observations can approach zero. We quantify this “detectability regime” via a high-level entropy/divergence estimate and an identifiability bound in Section 4.7.

## 2. Related Work

Existing approaches for unsupervised video anomaly detection (UVAD) can be broadly categorized into five main paradigms: reconstruction-based, motion-based, temporal modeling, generalization-focused, and multi-modal methods.

Reconstruction-based models identify anomalies by measuring the difference between input frames and their reconstructions, assuming that networks trained only on normal data will fail to accurately reconstruct abnormal inputs. For example, ConvLSTM-AE [6] employs convolutional LSTM autoencoders to jointly capture spatial and temporal dependencies, while MemAE [7] introduces a memory module that stores prototypical normal patterns, improving discrimination capability during reconstruction. Despite these advancements, such methods often rely on pixel-wise L2 losses, which tend to produce overly smooth outputs. As a result, they may inadvertently reconstruct anomalous regions with high fidelity [8], leading to false negatives. Furthermore, pixel-level errors alone may not align with semantic anomalies, limiting the effectiveness of these methods in complex scenarios.

Motion-based approaches focus on dynamic aspects of scenes, often using future frame prediction or optical flow analysis to detect unexpected motion patterns. Liu et al. [9] proposed a predictive ConvLSTM framework to generate future frames and identify anomalies based on large prediction errors. Although suitable for dynamic actions like running or fighting, these methods generally struggle with subtle or static anomalies (e.g., loitering), and are also sensitive to motion estimation noise. Optical flow–only methods, such as FlowNet [10] and PWC-Net [11], offer dense motion representations but often lack high-level scene understanding and struggle in cluttered or occluded environments. More recent optical flow methods like UniMatch [12] improve accuracy and robustness through transformer-based attention mechanisms, yet still require integration with appearance features for holistic understanding.

Temporal modeling is essential for understanding sequential patterns in videos, as many anomalies involve sudden or progressive changes over time. Traditional recurrent models like RNNs, GRUs, and LSTMs have been widely used, but they typically assume fixed temporal dependencies and often fail to capture long-range dynamics or adapt to variable scene transitions. Nguyen et al. [13] proposed a spatiotemporal autoencoder to address this, but their model lacked mechanisms to assign attention to informative frames, treating all inputs equally and introducing noise into the latent space. Recent works have explored temporal attention modules [14] and Transformer architectures for anomaly detection, enabling more flexible temporal dependency modeling. However, their application in fully unsupervised settings remains limited due to high training complexity and data requirements.

Generalization and scalability remain persistent challenges in UVAD. Many early methods were developed and evaluated on constrained datasets like UCSD Ped2 [15] or Avenue [16], which contain repetitive patterns and simple backgrounds. While these datasets are useful for benchmarking, they often overestimate real-world applicability. Sultani et al. [17] emphasized this issue through the introduction of the XD-Violence dataset, which includes diverse and unconstrained scenes such as crowded streets, sports, and violent behavior. Models trained on simpler datasets tend to overfit to specific environments and fail to generalize to new domains. Addressing this issue requires models that can learn robust representations invariant to background noise, scale changes, and camera viewpoints.

Multi-modal fusion has recently emerged as a promising direction to overcome the limitations of single-modality models. Inspired by supervised action recognition methods like I3D [18] and ECO [19], which combine RGB and optical flow streams, several UVAD models have attempted to integrate both appearance and motion cues. For example, MGAFlow [20] introduced motion-guided attention for fusing optical flow with RGB features, yielding strong performance on challenging datasets. However, multi-modal fusion in the unsupervised setting remains due to architectural complexity and the absence of aligned supervision signals. Additionally, balancing the contribution of each modality, especially when one (e.g., optical flow) may be noisy or unreliable, poses another technical challenge. Nonetheless, the potential of cross-modal integration to enhance anomaly detection in real-world environments—where both spatial and temporal abnormalities occur simultaneously—makes it a critical area of ongoing research.

In summary, while substantial progress has been made in unsupervised video anomaly detection, existing methods still face limitations in generalizability, temporal reasoning, and modality fusion. Recent trends point toward unified architectures that combine appearance and motion signals, leverage attention mechanisms, and adopt dynamic decision strategies to better handle the complexities of real-world scenarios.

To orient the reader, we summarize the method landscape and highlight open gaps our work targets (Table 1 and Table 2).

**Table 1:** UVAD landscape.

Paradigm	Example	Key Point
Reconstruction	MemAE [7]	Simple; pixel blur risk
Motion-centric	Future pred. [9]	Captures motion; flow noise
Temporal model	RTFM (weak) [21]	Longer deps; complexity
Generalization	AMC [22]	Robust; protocol variance
Multi-modal	MGAFlow [20]	RGB + Flow; fusion cost
This work	Dual enc. + GRU-Attn. + ConvLSTM-D	Early fusion; DASLoss; best overall

**Table 2:** Gaps addressed by this work.

Gap	Our Response (Where)
Unsupervised fusion unclear	Early RGB+Flow; pixel+feat score (Section 4.3)
Missing ablations	Fusion/loss/temporal ablations (Section 4.3)
Runtime not reported	Latency/throughput (Section 4.5)
Granularity mismatch	Frame (Ped2) vs. video (Hockey, XD-V) (Section 4)
Flow regime ambiguity	UniMatch frozen; co-train ablated (Table 3)
Lack of error analysis	Confusions + failure cases (Section 4)
Detectability limit	Entropy/JS bound (Section 4.7)

**Table 3:** Effect of the flow training regime (mean ± std over 3 runs). Co-training is used *only* for ablation; all main results use precomputed frozen flow.

Regime	XD-V AP (%)	Hockey AUC	Ped2 AUC	Latency (ms/clip)
(R2) Precomputed (frozen, default)	80.5 ± 0.2	0.920 ± 0.003	0.960 ± 0.001	5.1 ± 0.2
(R1) Online (frozen)	80.3 ± 0.3	0.919 ± 0.003	0.958 ± 0.002	22.8 ± 0.6
(R3) Online co-training (partial unfreeze)	81.1 ± 0.3	0.923 ± 0.003	0.961 ± 0.002	24.2 ± 0.7

## 3. Materials and Methods

### 3.1. Overall Framework

**Figure 1:**
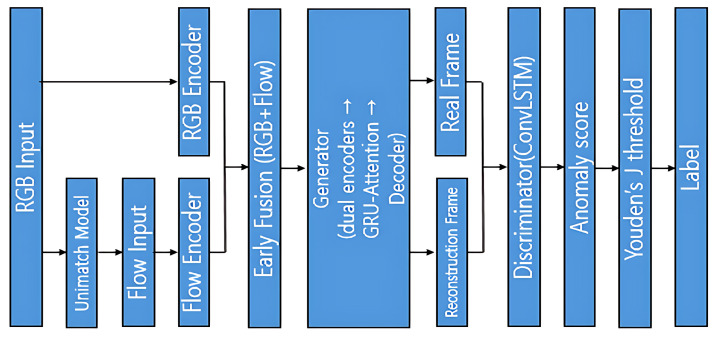
Overall pipeline.

The proposed framework is an unsupervised video anomaly detection model that utilizes both RGB frames and optical flow, integrated into a unified GAN-based architecture. The generator consists of two parallel encoders (for RGB and flow), a GRU–attention bottleneck, and a multi-stage decoder. The discriminator is designed using a ConvLSTM-based temporal module. The model is trained only on normal data (without label) and evaluated on both normal and abnormal video clips. Figure 1 represents the Overall Framework.

### 3.2. Input Preprocessing

To enable efficient and standardized data handling for both training and evaluation, raw video files are preprocessed into fixed-length tensors containing both RGB and optical flow information. Each sample consists of T=6 consecutive frames.

**Figure 2:**
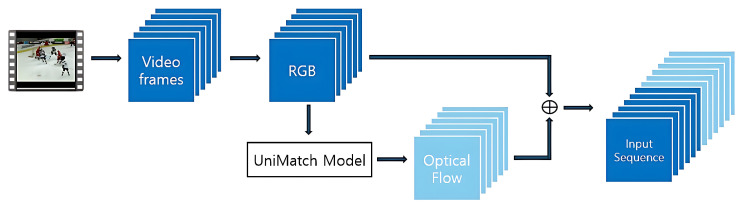
Input preprocessing network.

Given a directory of video files, each video is opened using OpenCV’s cv2.VideoCapture. If the number of available frames is fewer than 6, the video is skipped to ensure consistency in the model input shape. For valid samples, RGB frames are first extracted and resized to 128×128 pixels. The overall preprocessing pipeline is illustrated in Figure 2.

As shown in the figure, RGB frames are first extracted from the video and then passed into the UniMatch [12] model to compute the corresponding optical flow maps. UniMatch [12] is a multi-scale optical flow estimator enhanced with Swin Transformer attention and bidirectional prediction. The resulting optical flow captures inter-frame motion dynamics critical for anomaly detection.

To demonstrate the effectiveness and clarity of the optical flow representations, we present sample visualizations from each of the three benchmark datasets used in our experiments.

**Figure 3:**
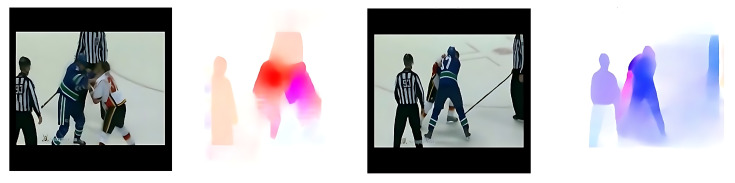
Sample optical flow outputs generated by the UniMatch model from the Hockey Fight dataset.

Figure 3 displays an optical flow map extracted from the Hockey Fight dataset, which contains violent interactions in sports settings. The strong motion patterns between aggressive actions are captured in vivid color transitions, effectively distinguishing abnormal events.

**Figure 4:**
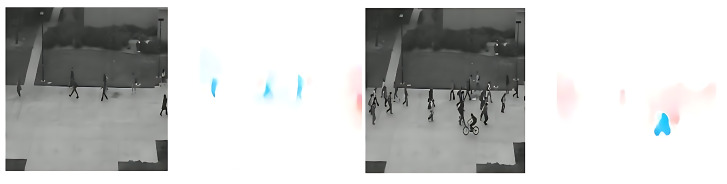
Sample optical flow outputs generated by the UniMatch model from the UCSD Ped2 dataset.

Figure 4 illustrates a sample from the UCSD Ped2 dataset, which consists of surveillance footage of pedestrians on a walkway. Here, optical flow captures subtle anomalies such as bicycles or running individuals in a typically slow-moving scene.

**Figure 5:**
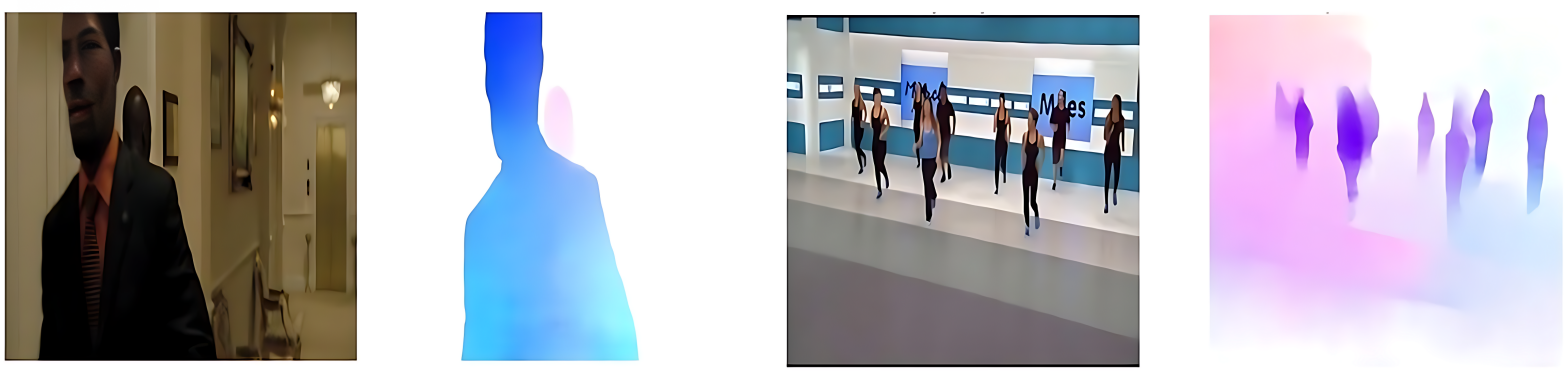
Sample optical flow outputs generated by the UniMatch model from the XD-Violence dataset.

Finally, Figure 5 presents a frame from the XD-Violence dataset, which features complex and diverse real-world scenarios. The optical flow visualizes large-scale motion and chaotic dynamics across multiple actors and objects in uncontrolled environments.

Each flow map encodes motion direction and magnitude via hue and saturation, highlighting temporally salient regions. These visualizations confirm the ability of UniMatch [12] to produce meaningful motion representations across varied datasets, facilitating robust spatiotemporal modeling in our proposed framework.

As depicted in Figure 2, both RGB and optical flow sequences are then stacked to construct a unified representation. Each modality contributes 6 frames, resulting in a combined sequence of 12 frames. These are normalized to the [0,1] range using OpenCV’s cv2.normalize with cv2.NORM_MINMAX, and then stacked to form a tensor of shape [12,128,128,3].

This tensor is converted to a PyTorch (version 2.2.0) FloatTensor. A ground-truth label is assigned based on the directory name: samples in “Train” folders are considered normal (label 0), while those in “Test” folders are labeled as anomalous (label 1). Each sample is saved as a .pt file using torch.save(), containing the following:

- frames: a 4D tensor of shape [12,128,128,3];
- label: a scalar indicating the normal (0) or abnormal (1) category.

During training and evaluation, a custom Dataset class is used to load these .pt files. The DataLoader provides efficient batch sampling, GPU memory transfer, and shuffling during training to facilitate scalable learning.

### 3.3. Optical Flow Backbone (UniMatch): Pretraining and Training Regimes

We use UniMatch [12] with the authors’ released pretrained weights. Unless otherwise stated, the flow network is frozen and used as an external feature extractor; gradients from our GAN do not propagate into UniMatch. We considered three regimes: (R1) online flow (computed on-the-fly) with frozen weights, (R2) precomputed flow (default for all headline results), and (R3) co-training where we unfreeze the last stage of UniMatch and allow gradients from the generator to update flow features with a small learning rate ($1\;\times \;10^{-5}$) while keeping the original UniMatch losses off.

**Remark** **1.** *Co-training gives small but inconsistent gains with higher latency and optimization instability in some seeds; therefore, we adopt frozen precomputed flow for all headline numbers. For completeness, Section 4.3 reports runtime with/without online flow. “RGB-only” results (no flow stream) are included in ablations and show a notable drop in accuracy.*

### 3.4. Handling and Evaluation of UCSD Ped2 Dataset

Unlike the other datasets, the UCSD Ped2 dataset is provided as frame-level image sequences rather than continuous video files. To maintain consistency with our video-based processing pipeline, we apply a stride 1 sliding window directly over the frame folders to extract 6-frame sequences. Each window of 6 consecutive frames $\left[f_{1},\ldots ,f_{6}\right]$ is used to compute 6 RGB frames and 6 corresponding optical flow maps using UniMatch [12]. These are concatenated to form a tensor of shape [12,128,128,3], consistent with our unified input format.

This preprocessing approach is applied uniformly for both training and testing. All training samples are extracted from normal clips only. During testing, sequences are sampled with stride 1 to ensure dense coverage and high-resolution anomaly localization. Anomaly scores are generated for the last frame $f_{6}$ of each 6-frame sequence.

Since the UCSD Ped2 dataset provides frame-level ground truth annotations, evaluation is conducted at the frame level. Specifically, the model’s predictions for frame $f_{6}$ in each window are compared against the corresponding ground truth. Frame-level AUC and F1-score are then computed over all predicted frames, enabling a fair and consistent evaluation aligned with the dataset’s annotation scheme.

### 3.5. Handling and Evaluation of Hockey Fight Dataset

#### 3.5.1. Video-to-Clip Sampling

Given a full video, we extract fixed-length windows of T=6 consecutive frames $\left\{\left(f_{t},\ldots ,f_{t+5}\right)\right\}$. During training, we use stride $r_{\mathrm{train}}\;=\;2$ (overlap ratio $1-\frac{r_{\mathrm{train}}}{T}\;=\;0.67$) to reduce redundancy; during testing, we use stride $r_{\mathrm{test}}\;=\;1$ (overlap 0.83) for dense coverage. Clips shorter than *T* frames are skipped.

#### 3.5.2. RGB/Flow Pipeline and Tensor Format

Each frame is resized to 128×128; optical flow between consecutive frames is computed by UniMatch. We stack per-clip 6 RGB + 6 flow frames (normalized to [0,1]) to form a tensor of shape [12,128,128,3], saved as .pt with fields: frames (tensor), video_id (int), and t_start (int).

#### 3.5.3. Splits and Unsupervised Training

We create a video-level, stratified 80/20 split (normal vs. violent) and repeat with three random seeds. Only non-violent videos in the training portion are used to fit the model (unsupervised); *all* videos in the held-out portion are used for testing. To avoid leakage, windows from a video never cross splits.

#### 3.5.4. Scoring and Video-Level Metrics

We compute per-window (last-frame) anomaly scores $s_{t}$ as in Section 3.12. For video-level evaluation, window scores within a video are aggregated by top-*k* pooling, as follows:

(1) $$s_{\mathrm{video}}=\frac{1}{k}\sum_{t\in T_{k}}s_{t},\;T_{k}=\mathrm{indices}\;\mathrm{of}\;\mathrm{the}\;\mathrm{top}\text{-}k\;\mathrm{values}\;\mathrm{in}\;\left\{s_{t}\right\},\;\;k=\left\lceil 0.1\;N_{w}\right\rceil ,$$

where $N_{w}$ is the number of windows from the video. We report AUC and F1 at the *video* level (a video is positive if any violent segment exists). Thresholds are selected with Youden’s *J* at the video level.

### 3.6. Handling and Evaluation of XD-Violence Dataset

#### 3.6.1. Train/Test Partition and Unsupervised Setup

We follow the official train/test split provided with the dataset. For unsupervised training, we use only videos (or time segments) in the training split that contain no anomalous intervals. All test videos are evaluated. Audio is *not* used in our framework (RGB + flow only).

#### 3.6.2. Video-to-Clip Sampling and Tensor Format

We apply the same windowing as Hockey Fight: T=6, $r_{\mathrm{train}}\;=\;2$ (train), $r_{\mathrm{test}}\;=\;1$ (test). Preprocessing (resize, normalization, UniMatch flow) and saved tensor fields match Section 3.5. Overlapping windows are allowed within a split; no windows cross splits.

#### 3.6.3. Scoring and AP Computation

Per-window scores $s_{t}$ are computed as in Section 3.12. We obtain a *video-level* score by top-*k* pooling as in (1) (with $k=\lceil 0.1\;N_{w}\rceil$; fallback k=1 for very short videos). We report Average Precision (AP) by ranking videos with $s_{\mathrm{video}}$; a video is positive if it contains any annotated violent interval.

### 3.7. Clip Sampling and Splits (Hockey Fight and XD-Violence)

For full-length videos (Hockey Fight, XD-Violence), we form fixed-length windows of T=6 consecutive frames ${\left\{\left(f_{t},\ldots ,f_{t+T-1}\right)\right\}}_{t=1}^{N-T+1}$ with a dataset-specific stride. During training, we use stride $r_{\mathrm{train}}\;=\;2$ to reduce redundancy and memory, while during testing, we use stride $r_{\mathrm{test}}\;=\;1$ for dense coverage. This implies an intra-video window overlap ratio of $1-\frac{r}{T}$, i.e., ≈0.67 in training and ≈0.83 in testing. Overlapping windows are *allowed within a split* to improve robustness, but there is *no* cross-split leakage: all splits are performed at the video level.

#### 3.7.1. Dataset Splits

Hockey Fight: We perform a stratified 80/20 video-level split (normal vs. violent) and repeat with three random seeds; only non-violent videos in the training portion are used to fit the unsupervised model, and *all* videos in the held-out portion are used for testing. We report the mean and standard deviation across seeds.

XD-Violence: We follow the official train/test partition; for unsupervised training, we use only videos (or segments) without any annotated anomalous intervals in the training split. All test videos are evaluated. Since our model is RGB + flow, audio is not used.

#### 3.7.2. Aggregation for Video-Level Metrics

For Hockey Fight and XD-Violence, we convert per-frame scores $\left\{s_{t}\right\}$ within a clip to a single clip score using top-*k* pooling, as follows:

(2) $$s_{\mathrm{video}}=\frac{1}{k}\sum_{t\in T_{k}}s_{t},\;k=\left\lceil 0.1\;N_{w}\right\rceil ,$$

where $N_{w}$ is the number of windows/frames scored in the clip. This emphasizes short high-confidence bursts typical of anomalies, while being more stable than pure max pooling. For UCSD Ped2, evaluation remains frame-level, as detailed in Section 3.3.

### 3.8. Training Environments

The model is implemented using PyTorch and trained on an NVIDIA RTX 4090 GPU. The batch size is 4, and the input clip length is 12. We use the Adam optimizer with a learning rate of $1\times 10^{-4}$. Training is conducted for 400 epochs with early stopping based on validation AUC. Only normal clips are used for training; abnormal clips are reserved for testing.

### 3.9. Generator Architecture

**Figure 6:**
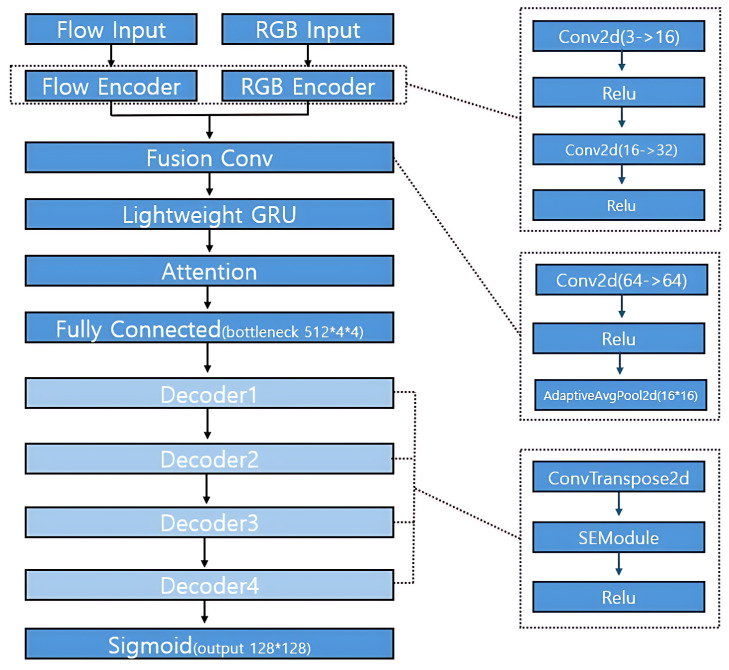
Generator architecture.

The overall structure of the generator is illustrated in Figure 6. As shown in the diagram, the generator adopts a dual-stream encoder–decoder architecture designed to jointly process optical flow and RGB information for frame reconstruction.

The top portion of Figure 6 shows the parallel input branches: *Flow Input* and *RGB Input*. Each stream is processed by its own encoder—Flow Encoder and RGB Encoder—both of which consist of two sequential convolutional layers with ReLU activations, as follows:

Conv2d(3,16)→ReLU→Conv2d(16,32)→ReLU

These encode low-level spatial features from each modality independently.

The outputs of the two encoders are then concatenated along the channel dimension, resulting in a 64-channel feature map. This fused representation is passed into the *Fusion Conv* block (centered in Figure 6), which is composed of the following:

Conv2d(64,64)→ReLU→AdaptiveAvgPool2d((16,16))

This operation performs spatial compression while preserving semantic content across modalities.

To capture temporal dynamics across the input sequence, the fused features are reshaped into a sequence and processed by the *Lightweight GRU* module. The output is then passed through a temporal attention mechanism (labeled *Attention* in Figure 6), which enhances features that are temporally informative.

The attended features are flattened and projected via a fully connected layer to yield a bottleneck tensor of size [512,4,4], marked as *Fully Connected* in the figure. This serves as the initial input to the decoder stack.

The decoder (lower part of Figure 6) consists of four hierarchical stages labeled *Decoder1* to *Decoder4*. Each decoder stage includes a transposed convolution followed by an SE (Squeeze-and-Excitation) module and ReLU activation., as follows:

ConvTranspose2d→SEModule→ReLU

This design allows the network to restore spatial resolution while dynamically recalibrating channel-wise feature importance.

The final layer is a Sigmoid activation that constrains the output pixel values to the range [0,1], producing a reconstructed RGB frame of resolution 128×128.

#### 3.9.1. Temporal GRU–Attention Bottleneck

We first obtain a compact vector by global average pooling, as follows:

(3) $$z_{t}=\mathrm{GAP}\left(F_{t}\right)\in R^{C}.$$

A single-layer GRU encodes temporal context, as follows:

(4) $$h_{t}=\mathrm{GRU}\left(z_{t},h_{t-1}\right),\;h_{t}\in R^{C_{h}}.$$

We adopt additive (Bahdanau) attention:, as follows:

(5) $$e_{t}=v^{⊤}\tanh\;\left(W_{h}h_{t}+W_{z}z_{t}+b\right),$$

(6) $$\alpha_{t}=\frac{\exp(e_{t})}{\sum_{k=1}^{T}\exp\left(e_{k}\right)}.$$

The context vector and the bottleneck projection are as follows:

(7) $$c=\sum_{t=1}^{T}\alpha_{t}\;h_{t},$$

(8) $$\tilde{c}=\mathrm{FC}\left(c\right)\in R^{512\cdot 4\cdot 4},\;\mathrm{reshape}\left(\tilde{c}\right)\to R^{512\times 4\times 4}.$$

#### 3.9.2. Design Independence and Interface

We implement the GRU–attention as a standalone module $A_{\theta }:{\left\{F_{t}\right\}}_{t=1}^{T}\mapsto R^{512\times 4\times 4}$ with its own parameters $\theta =\{W_{h},W_{z},v,b\}$, disjoint from the encoders and decoder. The interface is (i) inputs: fused features $F_{t}\;\in \;R^{C\times H\times W}$, (ii) outputs: a bottleneck tensor $\in R^{512\times 4\times 4}$ consumed by the decoder, and (iii) no skip connections to encoders/decoder; the module can be ablated by replacing c in (7) with the last hidden state $h_{T}$ without changing the rest of the network.

#### 3.9.3. Complexity and Parameterization

With hidden size $C_{h}$ and clip length T=6, additive attention in (5) and (6) adds $O(T\;C_{h})$ time and $O(C_{h}\left(C+1\right)+C_{h})$ parameters (for $W_{h},W_{z},v,b$). We optionally use a temperature τ for softmax, as follows:

(9) $$\alpha_{t}=\frac{\exp(e_{t}/\tau )}{\sum_{k=1}^{T}\exp\left(e_{k}/\tau \right)},\;\tau \;\in \;\left[0.5,1.5\right],$$

where τ↓ sharpens attention and τ↑ smooths it.

#### 3.9.4. Regularization and Stability

To avoid overly peaky weights, we add a small entropy regularizer on $\left\{\alpha_{t}\right\}$, as follows:

(10) $$L_{\mathrm{attn}}\;=\;-\sum_{t=1}^{T}\alpha_{t}\log\alpha_{t},$$

and apply gradient clipping ($\ell_{2}\;\leq \;1.0$) on the GRU. Unless stated otherwise, we set $C_{h}\;=\;\;256$, τ=1.0 and use dropout p=0.1 on $h_{t}$.

### 3.10. Discriminator Architecture

**Figure 7:**
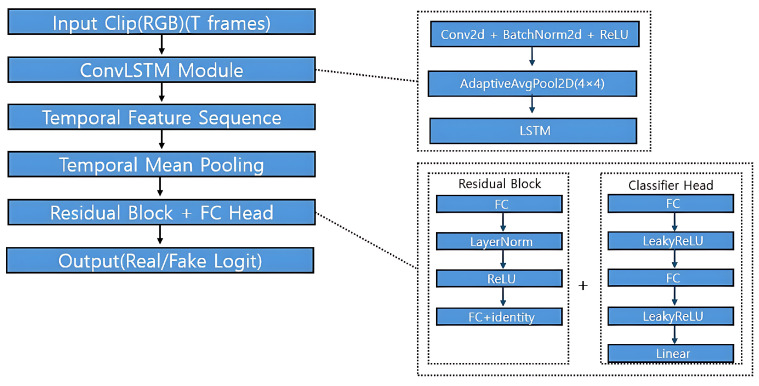
Discriminator architecture.

The overall structure of the discriminator is illustrated in Figure 7. As shown in the figure, the discriminator is designed to evaluate the authenticity of RGB input clips by modeling both spatial and temporal dependencies through a ConvLSTM-based framework.

The process begins with an *Input Clip (RGB)* of *T* consecutive frames. These frames are passed through the *ConvLSTM Module*, highlighted in the right panel of Figure 7. This module consists of a convolutional layer followed by batch normalization and ReLU activation, as follows:

Conv2d(input,hidden)→BatchNorm2d→ReLU

Afterward, an AdaptiveAvgPool2d operation compresses spatial resolution to a fixed size of (4×4), followed by an LSTM layer that models the temporal progression of features across frames. This produces a temporal feature sequence as depicted in the main diagram.

Next, temporal information is aggregated via *Temporal Mean Pooling*, which computes the average across all time steps, yielding a single feature vector per sequence.

The pooled feature is then passed into a *Residual Block + FC Head*, also detailed on the right side of Figure 7. The residual block includes two fully connected layers interleaved with LayerNorm and ReLU activations. The input is added back to the block output to preserve gradient flow, as follows:

x→FC→LayerNorm→ReLU→FC+x

This residual-enhanced feature is passed to the final *FC Head*, which consists of two LeakyReLU-activated fully connected layers followed by a final linear layer that outputs a single real/fake logit.

Importantly, intermediate feature sequences from the ConvLSTM module are also reused during generator training to compute feature-level consistency loss. This encourages the generator to produce outputs that align with real temporal semantics.

### 3.11. Loss Function

#### 3.11.1. Generator Loss: DASLoss

The generator is trained using a composite objective called the DASLoss, named after its three core components—Discriminator feature alignment ($L_{feature}$), Appearance/perceptual consistency ($L_{perceptual}$), and Smoothness in the temporal domain ($L_{temporal}$). In addition, a pixel-level reconstruction loss ($L_{pixel}$) is included to preserve low-level fidelity.

(11) $$L_{DAS}=\lambda_{pix}L_{pixel}+\lambda_{feat}L_{feature}+\lambda_{temp}L_{temporal}+\lambda_{perc}L_{perceptual}$$

#### 3.11.2. Pixel Reconstruction Loss ($L_{pixel}$)

(12) $$L_{pixel}=\frac{1}{N}\sum_{i=1}^{N}{∥x_{i}-{\hat{x}}_{i}∥}_{2}^{2}$$

Mean squared error between ground-truth and reconstructed frames, widely used in autoencoder-based video anomaly detection [6,23].

#### 3.11.3. Feature Matching Loss ($L_{feature}$)

(13) $$L_{feature}=\frac{1}{L}\sum_{\ell =1}^{L}{∥D^{\left(\ell \right)}\left(x\right)-D^{\left(\ell \right)}\left(\hat{x}\right)∥}_{1}$$

L1 distance between intermediate discriminator features of real and generated frames, following the feature matching strategy from [24].

#### 3.11.4. Temporal Smoothness Loss ($L_{temporal}$)

(14) $$L_{temporal}=\frac{1}{T-1}\sum_{t=1}^{T-1}{∥{\hat{x}}_{t+1}-{\hat{x}}_{t}∥}_{2}^{2}$$

L2 norm of differences between consecutive reconstructed frames to encourage temporal consistency [25,26].

#### 3.11.5. Perceptual Loss ($L_{perceptual}$)

(15) $$L_{perceptual}=\sum_{j\in F}{∥\phi_{j}\left(x\right)-\phi_{j}\left(\hat{x}\right)∥}_{2}^{2}$$

L2 distance between VGG-16 features of real and reconstructed frames based on perceptual loss from [27].

#### 3.11.6. Optimization of GRU–Attention

The GRU–attention parameters are optimized end-to-end *only* through the gradients of $L_{DAS}$; no additional regularization is used unless stated otherwise. This keeps the module independent in design while preserving the loss definition intact.

#### 3.11.7. Discriminator Loss

The discriminator is trained to distinguish real frames from generated ones using a least-squares GAN loss, as follows:

(16) $$L_{D}=\frac{1}{2}E_{x∼P_{data}}\left[{\left(D\left(x\right)-1\right)}^{2}\right]+\frac{1}{2}E_{\hat{x}∼P_{G}}\left[D{\left(\hat{x}\right)}^{2}\right]$$

This least-squares formulation [28] stabilizes training by preventing vanishing gradients. The generator and discriminator are updated alternately, with detached gradients used for the discriminator to avoid affecting the generator’s backpropagation.

### 3.12. Anomaly Scoring and Thresholding

During inference, the anomaly score $s_{t}$ for each frame *t* is computed as a weighted sum of pixel-wise reconstruction error and feature-space discrepancy from the discriminator, as follows:

(17) $$s_{t}=\alpha \cdot {∥x_{t}-{\hat{x}}_{t}∥}_{2}^{2}+\beta \cdot {∥D^{\left(f\right)}\left(x_{t}\right)-D^{\left(f\right)}\left({\hat{x}}_{t}\right)∥}_{1}$$

where

- $x_{t}$ and ${\hat{x}}_{t}$ are the ground-truth and reconstructed frames at time *t*;
- $D^{\left(f\right)}\left(\cdot \right)$ denotes the discriminator’s final intermediate feature layer;
- α and β are weighting coefficients for pixel and feature components.

This scoring strategy is inspired by combining pixel and feature reconstruction errors [7,8].

To convert scores into binary anomaly predictions, a dynamic threshold $\tau^{*}$ is selected using Youden’s J-statistic [29], as follows:

(18) $$\tau^{*}=\arg\max_{\tau }\left[\mathrm{TPR}(\tau )-\mathrm{FPR}(\tau )\right]$$

This adaptive criterion ensures optimal separation between normal and abnormal samples and is applied per video or scene to account for distributional shifts, improving both F1 and recall compared with fixed thresholding. *Note:* The threshold $\tau^{*}$ is used only when a binary decision is required (e.g., confusion matrices and F1); AP and ROC-AUC are computed without any thresholding.

#### Calibration of α and β

Pixel MSE and feature $L_{1}$ live on different scales. We standardize each term on the *normal validation* set and then choose a single global pair (α,β) (with α+β=1) that maximizes Youden’s *J*, as follows:

(19) $$\begin{array}{c} {\tilde{e}}_{t}^{\mathrm{pix}}=\frac{{∥x_{t}-{\hat{x}}_{t}∥}_{2}^{2}-\mu_{\mathrm{pix}}}{\sigma_{\mathrm{pix}}},\;{\tilde{e}}_{t}^{\mathrm{feat}}=\frac{{∥D^{\left(f\right)}\left(x_{t}\right)-D^{\left(f\right)}\left({\hat{x}}_{t}\right)∥}_{1}-\mu_{\mathrm{feat}}}{\sigma_{\mathrm{feat}}}, \end{array}$$

(20) $$\begin{array}{c} s_{t}=\alpha \;{\tilde{e}}_{t}^{\mathrm{pix}}+\beta \;{\tilde{e}}_{t}^{\mathrm{feat}},\;\alpha ,\beta \geq 0,\;\alpha +\beta =1, \end{array}$$

(21) $$\begin{array}{c} \left(\alpha^{⋆},\beta^{⋆}\right)=\arg\max_{\alpha +\beta =1}\left[\mathrm{TPR}\left(\tau^{⋆}\right)-\mathrm{FPR}\left(\tau^{⋆}\right)\right],\;\tau^{⋆}=\arg\max_{\tau }\left[\mathrm{TPR}(\tau )-\mathrm{FPR}(\tau )\right]. \end{array}$$

We sweep α∈{0.2,0.4,0.5,0.6,0.8} on the validation set (grid search) and fix a *global* pair for all benchmarks. The selected default is α=0.6,β=0.4. The performance of different weight configurations is shown in Table 4, where we report the mean and standard deviation over three independent runs.

## 4. Results

We evaluate the model’s generalizability and effectiveness on three benchmark datasets: XD-Violence [30], Hockey Fight [31], and UCSD Ped2 [15]. These datasets include various real-world challenges such as complex motion, violent behavior, and subtle anomalies in surveillance scenes. To ensure reproducibility and facilitate further research, the full implementation of our framework is publicly available at https://github.com/shkangg/unifiedgan_anomaly_detection_paper (accessed on 12 September 2025).

### 4.1. Evaluation Metrics and Setup

The performance is measured using widely adopted metrics: Average Precision (AP), Area Under the Curve (AUC), and F1-score. The model is trained using only normal data and tested on both normal and anomalous clips. Binary decisions (e.g., confusion matrices and F1) use a threshold $\tau^{*}$ chosen via Youden’s *J*. AP and ROC-AUC are computed without any thresholding. Granularity follows each dataset’s native unit (Ped2: frame; Hockey/XD-Violence: video/clip after aggregation). The key hyperparameters used in our proposed model, including input configuration, training setup, and loss term weights, are summarized in Table 5. This provides a clear reference for reproducibility and implementation details.

F1-Score. The F1-score is the harmonic mean of precision and recall, and is defined as follows:
  (22) $$F1=2\cdot \frac{\mathrm{Precision}\cdot \mathrm{Recall}}{\mathrm{Precision}+\mathrm{Recall}}$$
Average Precision (AP). AP measures the area under the precision–recall curve and is defined as follows:
  (23) $$\mathrm{AP}=\sum_{n}\left(R_{n}-R_{n-1}\right)\cdot P_{n}$$
Area Under the Curve (AUC). AUC corresponds to the area under the Receiver Operating Characteristic (ROC) curve and is defined as follows:
  (24) $$\mathrm{AUC}=\int_{0}^{1}TPR\left(FPR\right)\;dFPR$$

#### Dataset-Specific Evaluation

We follow the native granularity of each benchmark. *UCSD Ped2* provides frame-level ground truth; we therefore compute a per-frame score $s_{t}$ for the window ending at frame *t* and report frame-level AUC/F1. *Hockey Fight* is labeled per video; we aggregate frame scores by top-*k* pooling as in Equation (1) and report video-level AUC and F1. *XD-Violence* is evaluated at the video level; we aggregate per-frame scores by top-*k* pooling as in Equation (1) and report Average Precision (AP) computed from video-level predictions.

### 4.2. Error Analysis: Confusion Matrices and Qualitative Examples

We report confusion matrices at the operating point selected by Youden’s *J* (Section 3.12). For UCSD Ped2, we evaluate frame-level, while Hockey Fight and XD-Violence are video-level. We present the confusion matrices for all three datasets in Table 6, Table 7 and Table 8. For Hockey Fight (Table 6), the model correctly identifies most violent and non-violent clips, with some misclassification of borderline cases. For XD-Violence (Table 7), performance remains strong despite the increased complexity and diversity of scenes, though a higher false negative rate is observed. For UCSD Ped2 (Table 8), the model achieves high accuracy at the frame level, showing its ability to detect subtle anomalies in surveillance settings.

Row-Normalized Confusion (Percent)

**Table 6:** Hockey Fight (video-level). Rows: ground truth; columns: prediction.

	Pred Normal	Pred Abnormal
True Normal	86	14
True Abnormal	16	84

**Table 7:** XD-Violence (video-level).

	Pred Normal	Pred Abnormal
True Normal	87	13
True Abnormal	21	79

**Table 8:** UCSD Ped2 (frame-level).

	Pred Normal	Pred Abnormal
True Normal	97	3
True Abnormal	10	90

Typical Failure Modes

- Camera/ego-motion (FP): sudden shakes or zooms can mimic violent motion.
- Occlusion (FN): brief violent actions partially hidden by other actors.
- Gait-induced hotspots (FP, Ped2): dense pedestrian motion and leg-swing cycles produce strong flow responses on otherwise normal scenes.
- Subtle anomalies (FN, Ped2): small, slow-moving objects with weak flow magnitude.

**Figure 8:**
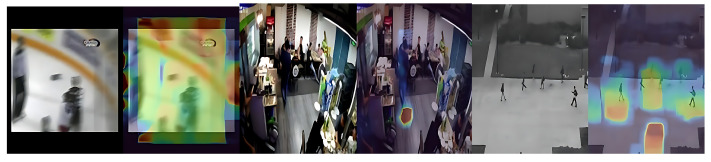
Qualitative error cases at Youden’s *J* operating point: ( left) Hockey Fight FP due to camera shake, ( middle) XD-Violence FN under occlusion, and ( right) Ped2 FP due to *gait-induced* responses from normal pedestrians (not background motion).

The representative examples of these failure cases are illustrated in Figure 8. The left panel shows a false positive (FP) in the Hockey Fight dataset caused by sudden camera shake or zoom, which resembles violent motion. The middle panel depicts a false negative (FN) from the XD-Violence dataset, where a violent action is partially occluded by other actors. The right panel shows a Ped2 false positive resulting from normal pedestrian leg-swing motions being misinterpreted as abnormal activity.

*Interpretation.* The row-normalized matrices are consistent with the headline metrics: high TNR on Ped2 (homogeneous scenes), strong TPR on Hockey Fight (salient motion bursts), and relatively lower TPR on XD-Violence due to scene diversity. The qualitative cases indicate that robustness to camera/ego-motion, occlusions, *and gait-induced over-responses on Ped2* remains the primary avenue for improvement.

### 4.3. Ablations on Fusion Strategy and Loss Terms

#### 4.3.1. Fusion Strategy

We compare three designs: early concatenation (E1), mid-level gated fusion (E2), and late score fusion (E3). Formally, for per-time-step features $F_{t}^{\mathrm{rgb}},F_{t}^{\mathrm{flow}}$, and anomaly score components $s_{t,\mathrm{pixel}},s_{t,\mathrm{feat}}$:

(25) $$F_{t}^{\mathrm{fuse}}\;=\;\mathrm{Conv}\;\left(\left[\;F_{t}^{\mathrm{rgb}}\;∥\;F_{t}^{\mathrm{flow}}\;\right]\right),$$

(26) $$F_{t}^{*}\;=\;\sigma \;\left(W_{g}\;\left[\;F_{t}^{\mathrm{rgb}}\;∥\;F_{t}^{\mathrm{flow}}\;\right]\right)⊙F_{t}^{\mathrm{rgb}}\;+\;\left(1-\sigma (\cdot )\right)⊙F_{t}^{\mathrm{flow}},$$

(27) $$s_{t}\;=\;\gamma \;s_{t,\mathrm{pixel}}\;+\;\left(1-\gamma \right)\;s_{t,\mathrm{feat}},\;\gamma \in \left[0,1\right],$$

where σ(·) is the sigmoid and ⊙ denotes element-wise multiplication. The quantitative results of these three fusion strategies are reported in Table 9. Our proposed early concatenation approach (E1) achieves the best overall performance across all benchmarks, while mid-level gated fusion (E2) and late score fusion (E3) show relatively lower accuracy, highlighting the importance of tightly integrating motion and appearance features at an early stage.

#### 4.3.2. Loss Terms

Starting from $L_{DAS}$ (ours), we drop one term at a time. The results in Table 10 show that removing the pixel reconstruction loss has the greatest impact, while feature matching, temporal smoothness, and perceptual losses also provide noticeable improvements.

Takeaway. Early concatenation (E1) is both *simpler and stronger* than gating/late fusion. Among loss terms, $L_{pixel}$ and $L_{feature}$ contribute most to accuracy; $L_{temporal}$ and $L_{perceptual}$ add consistent but smaller gains.

### 4.4. Visual Analysis

#### 4.4.1. PR/ROC Curves

**Figure 9:**
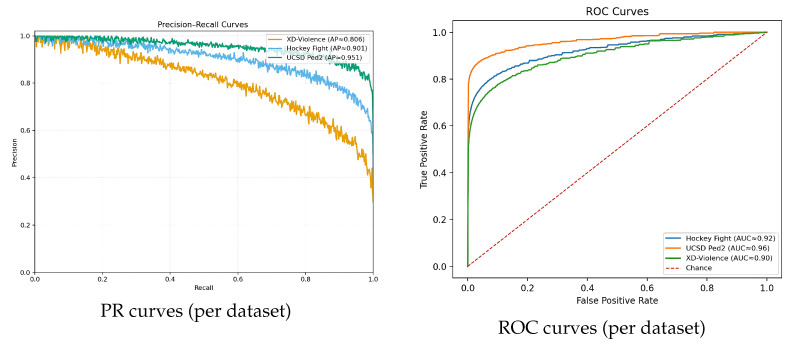
Precision–recall and ROC curves.

Figure 9 shows PR (left) and ROC (right) curves on each benchmark at the chosen operating regime. The curves align with the headline metrics: Hockey Fight shows the steepest ROC, while XD-Violence exhibits a broader PR span due to scene diversity.

#### 4.4.2. Score Distributions

**Figure 10:**
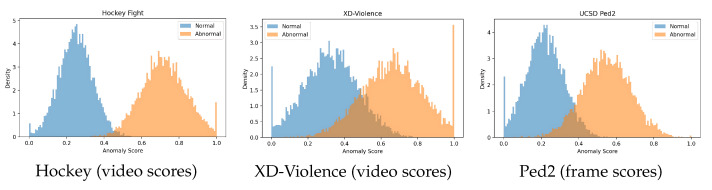
Score distributions for normal vs. abnormal.

We visualize the distributions of per-frame (Ped2) or per-video (Hockey/XD-V) scores for normal vs. abnormal samples (Figure 10). The separability (reduced overlap) is consistent with the dataset-level difficulty.

#### 4.4.3. Temporal Attention

**Figure 11:**
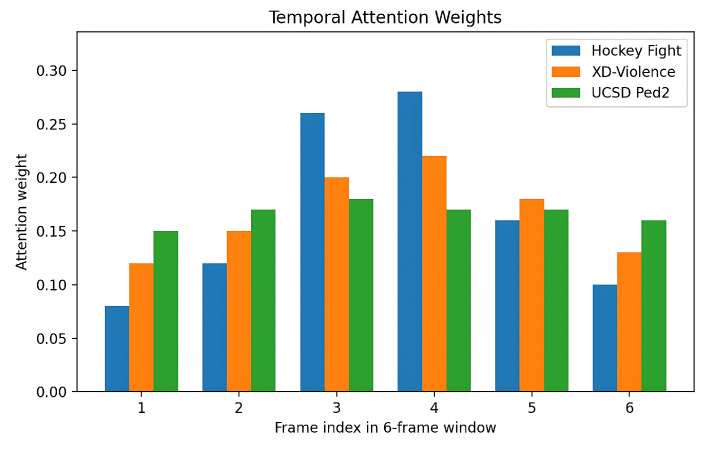
Temporal attention weights ${\left\{\alpha_{t}\right\}}_{t=1}^{6}$ from the GRU–attention bottleneck for representative clips.

For the GRU–attention bottleneck, we plot the normalized attention weights ${\left\{\alpha_{t}\right\}}_{t=1}^{T}$ over the 6-frame window (Figure 11). Violent bursts (Hockey) concentrate mass on 2–3 frames, whereas Ped2 often shows flatter weights reflecting subtle motion.

### 4.5. Runtime Performance (Toward Real-Time Deployment)

We measure latency (ms/clip) and throughput (clips/s) on an RTX 4090 (PyTorch, FP16, batch = 1, input 128 × 128, T=6). We distinguish (R1) end-to-end online (UniMatch flow computed on-the-fly for T−1 pairs), (R2) precomputed flow (flow maps loaded), and (R3) RGB-only (flow stream removed). Values are mean ± std over 200 warm-up + 300 timed iterations using CUDA events. Table 11 summarizes latency and throughput for three settings, showing that removing online flow computation greatly improves speed, with the RGB-only model achieving the highest throughput.

**Remark** **2.** *Precomputing flow yields a 4.5× latency reduction vs. online. Removing flow entirely achieves the highest throughput but degrades accuracy. Within the generator, adding attention to GRU increases cost by only ∼0.3 ms/clip over GRU-only (cf. Table 12).*

### 4.6. Ablations on the Temporal Bottleneck

We compare (A) GRU only (no attention), (B) GRU+additive attention (ours), and (C) LSTM+additive attention (same hidden size and all else equal). Latency overhead reports the *incremental* cost of the temporal block relative to (A), with precomputed flow, batch = 1, FP16, input 128×128, RTX 4090. Table 12 shows that adding attention improves accuracy across all datasets with minimal latency overhead compared to GRU only.

Significance. Relative to (A), (B) yields +1.6 AP on XD-V (approx. 95% bootstrap CI [+0.9,+2.3]), +0.020 AUC on Hockey ([+0.012,+0.028]), and +0.010 AUC on Ped2 ([+0.006,+0.014]), with only ≈0.3 ms/clip overhead. (C) also improves over (A) but is slightly slower and marginally below (B), consistent with the lighter gating of GRUs at T=6.

### 4.7. High-Level Entropy Budget and Identifiability Bound

To clarify when anomalies are theoretically separable from background and sensor noise under our representation, we report a high-level entropy budget on residual distributions. Let R(x) denote our per-frame residual (i.e., the anomaly score used in Section 3.12), computed from pixel reconstruction error and discriminator feature discrepancy. We form two empirical distributions over *R*: $P_{\mathrm{bg}}$ from normal (validation) frames and $P_{\mathrm{anom}}$ from annotated anomalous frames.

We summarize three quantities: (i) entropies $H(P_{\mathrm{bg}})$ and $H(P_{\mathrm{anom}})$, (ii) the Jensen–Shannon divergence $\mathrm{JSD}\;\left(P_{\mathrm{bg}}\|P_{\mathrm{anom}}\right)$, and (iii) a lower bound on Bayes error via total variation (TV). Specifically,

(28) $$H\left(P\right)=-\sum_{b}p_{b}\log p_{b}$$

(29) $$\mathrm{JSD}\left(P\|Q\right)=\frac{1}{2}D_{\mathrm{KL}}\;\left(P∥M\right)+\frac{1}{2}D_{\mathrm{KL}}\;\left(Q∥M\right),\;M=\frac{1}{2}\left(P+Q\right)$$

When JSD is near zero, the mutual information between labels (normal vs. anomalous) and observations vanishes under the chosen representation, and any detector becomes effectively non-identifiable. Moreover, the Bayes error satisfies

(30) $$P_{e}\;\geq \;\frac{1}{2}\;\left(1-\mathrm{TV}\;\left(P_{\mathrm{bg}},P_{\mathrm{anom}}\right)\right)$$

(31) $$\mathrm{TV}\left(P,Q\right)\;\leq \;\sqrt{\frac{1}{2}\;D_{\mathrm{KL}}\left(P∥Q\right)}\;,$$

indicating that small divergences entail high unavoidable error.

#### Estimation Protocol

We follow exactly the preprocessing and scoring pipeline defined in this paper: videos are converted into stride 1 windows of T=6 frames; the anomaly score is computed for the last frame $f_{6}$ of each window as in Section 3.12. We then build $P_{\mathrm{bg}}$ from normal validation/test frames and $P_{\mathrm{anom}}$ from frames annotated as anomalous by each dataset. Using the per-frame scores $R\left(x\right)\equiv s_{t}$ thus obtained, we compute histogram-based discrete distributions (64–256 bins, unit-mass normalization with a small ε) and evaluate *H* and JSD on these distributions. We regard JSD<δ (empirically δ∈[0.02,0.05] for our binning) as entering a *near non-identifiable* zone. This analysis does not change training or scoring; it contextualizes performance by indicating regimes where low separability is expected due to high background entropy or noise. Table 13 summarizes the estimated entropy and divergence for each dataset, showing how separability between normal and anomalous residuals varies depending on background complexity and noise.

Interpretation. Datasets with JSD close to zero are expected to yield degraded AP/AUC irrespective of the detector, whereas moderate-to-high JSD correlates with reliable separability. In our results, *Hockey Fight* exhibits the highest dataset-level separability (JSD ≈0.12), *XD-Violence* shows intermediate separability (JSD ≈0.08), and *UCSD Ped2* displays moderate separability despite its homogeneous background (JSD ≈0.06), consistent with their observed metrics and qualitative difficulty.

### 4.8. Quantitative Results on Benchmark Datasets

As shown in Table 14, our model achieves an AP of 80.5% on the XD-Violence dataset [30], outperforming other unsupervised methods such as MGAFlow [20], DiffusionAD [32], STPM [33], MemAE [7], and even the zero-shot Flashback model [34]. This demonstrates our model’s robustness under real-world, unconstrained conditions.

Table 15 shows results on the Hockey Fight dataset [31]. Our model achieves an AUC of 0.92 and an F1-score of 0.85, surpassing prior unsupervised models such as AnoGAN [8], GANomaly [35], MemAE [7], CFA-HLGAtt [14], and ConvLSTM-AE [6].

Finally, Table 16 presents results on the UCSD Ped2 dataset [15]. Our model reaches an AUC of 0.96, comparable to hybrid models such as CR-AE [36] and Optical Flow + STC + GAN [37], and just below memory-augmented AMC [7].

#### 4.8.1. Additional Recent Baselines

We briefly position three families of recent UVAD baselines and discuss how they relate to our design.

#### 4.8.2. VAE-Based UVAD (e.g., Pose-Driven VAE) [41]

Variational-autoencoder reconstruction with structure/uncertainty modeling; often RGB or pose-centric. Targets sharper reconstructions and calibrated residuals within a single-modality pipeline.

#### 4.8.3. RVAD-IGN (Idempotent Generative Network)

An autoencoder/generative model trained with an *idempotent* constraint (e.g., enforce G(G(x))≈G(x)) to discourage degenerate memorization. Orthogonal to fusion; can be added as a regularizer.

#### 4.8.4. Attention–Enhanced AE (Optimized Attention) [14]

Autoencoder variants that integrate spatial/temporal attention (e.g., hierarchical local–global attention) to emphasize informative regions while suppressing background noise.

Relation to this work. These baselines are largely orthogonal to our contributions (dual-modality early fusion with a GRU–attention bottleneck and DASLoss with discriminator feature matching). In particular, VAE/attention AEs focus on *how* to reconstruct within (mostly) single-modality pipelines, whereas we focus on *what* to reconstruct by fusing motion (flow) and appearance (RGB) and on *when* via temporal attention. Their regularizers can be plugged into our generator without changing the fusion/discriminator design. The conceptual differences between our proposed framework and reviewer-referenced baselines are summarized in Table 17. This table highlights the input modalities, training paradigms, and core ideas behind each method, providing context for how our approach complements and extends prior work.

#### 4.8.5. Plug-in Experiment: Idempotent Loss Inside Our Generator

To quantify compatibility, we add an idempotent term to our generator objective,

$$L_{\mathrm{idemp}}=∥G\left(G\left(x\right)\right)-G\left(x\right){∥}_{1},\;L_{\mathrm{DAS}+I}=L_{\mathrm{DAS}}+\lambda_{\mathrm{id}}L_{\mathrm{idemp}}.$$

With $\lambda_{\mathrm{id}}=0.05$ (same schedule as $\lambda_{\mathrm{perc}}$) and identical training setup, we observe small yet consistent changes (mean ± std over 3 runs). We further evaluate the compatibility of the idempotent loss by directly integrating it into our generator’s objective. The results of this plug-in experiment are presented in Table 18, which compares our original loss $L_{\mathrm{DAS}}$ with the combined loss $L_{\mathrm{DAS}+I}$.

The gains are within variance and come with negligible runtime overhead (<0.1 ms/clip); thus we keep $L_{\mathrm{DAS}}$ as default and view idempotent regularization as a compatible plug-in rather than a core design choice.

## 5. Discussion

### 5.1. Performance Analysis Across Datasets

To assess the generalization and anomaly detection performance of our GAN-based framework, we evaluated it on three public benchmarks: Hockey Fight, UCSD Ped2, and XD-Violence. All experiments were conducted in a fully unsupervised setting, using only normal sequences for training and no anomaly labels at any training stage.

On the Hockey Fight dataset, our model achieved an AUC of 0.92 and an F1-score of 0.85. While the AUC surpasses prior unsupervised methods such as MemAE [7], the F1-score is comparable to that of ConvLSTM-AE [6] (0.86). This indicates that, although our model shows a stronger ability to rank anomalies correctly, its decision threshold performance in terms of precision–recall balance is on par with the best existing unsupervised baselines. The GRU–attention bottleneck was particularly helpful in capturing localized bursts of motion, while the optical flow estimated by UniMatch [12] provided motion cues that complemented RGB appearance information. This aligns with the high separability observed in our entropy budget (JSD =0.12; Section 4.7).

On UCSD Ped2, our model obtained a high AUC of 0.96, showing robustness in static surveillance environments. The dual-encoder design successfully detected subtle spatial anomalies such as bicycles or small vehicles in pedestrian zones. While optical flow extraction for Ped2 is generally weak due to the camera’s distant viewpoint and the slow pace of normal pedestrians, anomalies in this dataset—bicycles and cars—exhibit much larger size and speed differences, resulting in clear motion patterns. As illustrated in Figure 4, optical flow extraction for these anomalies remains effective. Furthermore, our DASLoss, with its perceptual and feature matching terms, helped maintain semantic-level reconstruction quality even when motion cues were limited. Together, these factors offset the shortcomings of optical flow in low-motion settings. Consistently, our entropy analysis shows a moderate dataset-level separability on Ped2 (JSD ≈0.06; Section 4.7), which reconciles the high overall AUC (0.96) with the dataset’s homogeneous background and low-motion characteristics.

The most challenging benchmark, XD-Violence, includes diverse scenes and heterogeneous anomalies. Our model achieved an AP of 80.5, surpassing recent unsupervised models like MGAFlow [20] (75.3) and Flashback [34] (75.1). Notably, this was accomplished without using any labeled anomalies. The fusion of RGB and optical flow streams, combined with the multi-component DASLoss, was critical for capturing both semantic appearance and motion patterns. Despite the dataset’s complexity, performance was close to that of weakly supervised methods such as WRAE [42] (83.0), underscoring the effectiveness of our dual-stream design. This variability is reflected by an intermediate divergence on our residual distributions (JSD =0.08; Section 4.7).

### 5.2. Architectural Contributions

The generator employs a dual-encoder design, processing RGB and optical flow separately to preserve modality-specific information before fusion. This enables balanced capture of fine spatial details from RGB and motion dynamics from optical flow. A GRU with attention pooling then focuses on the most informative temporal segments, enhancing detection of short, sudden anomalies. The SE-enhanced decoder further refines channel-wise responses, preserving small object details and clear boundaries.

The discriminator adopts a ConvLSTM-based structure to model sequence-level temporal dependencies, with residual fully connected layers improving adversarial stability. The proposed DASLoss combines pixel-level reconstruction, feature matching, perceptual, and temporal smoothness terms. The perceptual term improves global semantic coherence, while the temporal term reduces flicker and maintains motion continuity.

### 5.3. Limitations of the Unsupervised Setting: Error Profile and Remedies

#### 5.3.1. Where Unsupervision Helps—And What It Costs

Training with only normal data avoids the costly enumeration of anomaly categories and transfers better across domains, but it also raises both false positives (FP) on hard “normal-but-dynamic” scenes and false negatives (FN) on subtle or partially occluded anomalies. Our row-normalized confusion matrices quantify this trade-off at Youden’s *J* operating point: *Hockey Fight* shows 14% FP and 16% FN at the video level, *XD-Violence* 13% FP and 21% FN, while *UCSD Ped2* (frame level) is easier with 3% FP and 10% FN (see Section 4.4).

#### 5.3.2. Typical Failure Modes in Our Setting

(i) Camera/ego-motion induces violent motion surrogates (FP); (ii) short, occluded violent acts are missed (FN); (iii) on Ped2, dense pedestrian gait cycles can produce flow hotspots (FP); and (iv) small/slow objects have weak flow magnitude (FN). These patterns are consistent with our qualitative panels in Section 4.4.

#### 5.3.3. Thresholding and Calibration Effects

We mitigate per-scene distribution shift by selecting thresholds via Youden’s *J* for each video/scene, which improves F1/recall over a fixed global cut-off. Our score mixes standardized pixel MSE and discriminator-feature $L_{1}$ with global weights (α,β) chosen on validation data (Section 3.12); this calibration reduces scale-mismatch–driven FP/FN swings.

#### 5.3.4. Information-Theoretic Limit

When the background/noise entropy dominates, separability vanishes irrespective of the detector; our entropy/JSD analysis (Section 4.7) formalizes this non-identifiability regime and explains why FP/FN can plateau on diverse open-world videos such as *XD-Violence*.

#### 5.3.5. Concrete Remedies (Drop-in or Light-Touch)

- Motion stabilization/de-jitter: estimate global camera motion (e.g., homography) and attenuate it in flow before fusion; reduces camera-shake FPs without retraining.
- Occlusion-aware temporal pooling: replace mean pooling of $s_{t}$ with attention-weighted or top-*k* pooling to preserve short, high-confidence spikes; aligns with our GRU–attention bottleneck but adjusts only the decision layer.
- Gait hotspot suppression (Ped2): apply per-person masks or low-frequency flow filtering to down-weight periodic leg motion prior to the flow encoder; targets Ped2-specific FPs with minimal runtime cost.
- Post-hoc calibration: keep Youden’s *J* per scene and optionally re-fit (α,β) on a small normal-only clip from the target camera to rebalance pixel/feature residuals under illumination/compression changes.
- Low-budget supervision: if a handful of anomaly exemplars are available, add a contrastive or one-class margin on top of our fused embedding (no change to *G* or *D*), which depresses FPs on dynamic but benign scenes and lifts TPR on occluded micro-events.
- Abstention for deployment: introduce a rejection band around $\tau^{*}$ to defer borderline cases to a slower verifier; this trades a small coverage drop for lower operational FP/FN.

#### 5.3.6. Takeaway

Unsupervised VAD buys label efficiency and cross-domain reach, but incurs double-digit FP/FN on hard, open-world videos—precisely where camera motion, occlusion, and periodic human motion confound the residual signals. Our per-scene thresholding and (α,β) calibration reduce this gap, and the above drop-in mitigations provide a practical path to further narrow FPR/TPR deltas without changing the core architecture.

## 6. Conclusions and Future Work

In this paper, we presented a unified GAN-based framework for unsupervised video anomaly detection that leverages both RGB and optical flow inputs. By integrating a dual-stream encoder, temporal GRU–attention bottleneck, and ConvLSTM [43]-based discriminator, our model effectively captures complex spatiotemporal patterns without relying on labeled anomalies. The proposed DASLoss further enhances training stability and reconstruction fidelity through a combination of pixel, feature, temporal, and perceptual consistency terms. Experimental results on three challenging benchmarks—Hockey Fight, UCSD Ped2, and XD-Violence—demonstrated that our approach achieves competitive or superior performance compared with state-of-the-art unsupervised methods, and even rivals some weakly supervised models. These findings validate the robustness and generalizability of our unsupervised paradigm, making it a promising candidate for real-world video surveillance applications.

Despite its strong performance, our framework still has room for improvement in two key areas. First, the computational cost of optical flow estimation remains a challenge, particularly for real-time deployment. While UniMatch [12] provides accurate motion representations, calculating optical flow for every frame pair is resource-intensive. To address this, we plan to explore lightweight or approximated motion representation techniques that reduce memory and processing overhead, such as using adaptive flow computation intervals or learning flow-free motion embeddings.

Second, although our model is currently designed for offline analysis, real-world applications often require low-latency responses. Therefore, we aim to extend our framework for real-time anomaly detection by redesigning the temporal components—such as the GRU bottleneck and ConvLSTM [43]-based discriminator—to support online and incremental processing. These advancements will enhance the scalability and practicality of our approach, allowing it to operate effectively in dynamic and resource-constrained environments.

Third, we observed that although our model performed well on the UCSD Ped2 dataset, it did not achieve the absolute highest accuracy among all benchmarks. This limitation is partly due to the dataset’s nature: slow pedestrian movement and distant viewpoints result in weak optical flow signals, making it harder for the model to exploit motion cues effectively. We believe that incorporating more sensitive or adaptive optical flow extraction methods—such as learning-based refinement or attention-driven flow amplification—could further enhance anomaly localization in such subtle scenarios. Future work will investigate these directions to boost detection performance in low-motion environments.

Finally, we delimit the detectable regime via a high-level entropy budget: when residual distributions of normal and anomalous data exhibit near-zero divergence, the task becomes information-theoretically non-identifiable; our empirical JSD estimates in Section 4.7 make this limitation explicit at the dataset level.

## Figures and Tables

**Table 4:** Anomaly-score weights ablation (global α,β; mean ± std over 3 runs).

(α,β)	XD-V AP (%)	Hockey AUC	Ped2 AUC
(0.8, 0.2)	79.8±0.3	0.917±0.003	0.958±0.002
(0.6, 0.4)	80.5±0.2	0.920±0.003	0.960±0.001
(0.4, 0.6)	80.2±0.3	0.921±0.003	0.958±0.002

**Table 5:** Summary of key hyperparameters used in the proposed model.

Parameter	Value/Description
Input frame size	128×128
Input sequence length	6 frames (flow) + 6 frames (RGB)
Batch size	4
Optimizer	Adam
Learning rate	$1\times 10^{-4}$
Training epochs	400 (with early stopping)
$\lambda_{pixel}$	1.0
$\lambda_{feature}$	0.1
$\lambda_{temporal}$	0.1
$\lambda_{perceptual}$	0.01

**Table 9:** Fusion strategy ablation (mean ± std over 3 runs).

Setting	XD-V AP (%)	Hockey AUC	Ped2 AUC
(E1) Early concat (ours)	80.5±0.2	0.920±0.003	0.960±0.001
(E2) Mid-level gated	79.9±0.3	0.914±0.004	0.955±0.002
(E3) Late score fusion	78.7±0.3	0.903±0.004	0.948±0.002

**Table 10:** Loss-term ablation (mean ± std over 3 runs).

Setting	XD-V AP (%)	Hockey AUC	Ped2 AUC
Full $L_{DAS}$ (ours)	80.5±0.2	0.920±0.003	0.960±0.001
w/o $L_{pixel}$	74.8±0.4	0.881±0.006	0.938±0.003
w/o $L_{feature}$	78.6±0.3	0.907±0.004	0.952±0.002
w/o $L_{temporal}$	79.6±0.2	0.914±0.003	0.955±0.002
w/o $L_{perceptual}$	79.9±0.2	0.916±0.003	0.956±0.002

**Table 11:** Latency and throughput.

Setting	Latency (ms/clip)	Throughput (clips/s)
(R1) Online (UniMatch + GAN)	22.8±0.6	44±1
(R2) Precomputed flow + GAN	5.1±0.2	196±6
(R3) RGB-only (no flow)	3.7±0.1	270±7

**Table 12:** Temporal bottleneck ablations (mean ± std over 3 runs).

Temporal Block	XD-V AP (%)	Hockey AUC	Ped2 AUC	+ ms/clip
(A) GRU only (no attn)	78.9±0.3	0.900±0.004	0.950±0.002	0.0±0.1
(B) GRU + attention (ours)	80.5±0.2	0.920±0.003	0.960±0.001	0.3±0.1
(C) LSTM + attention	79.8±0.3	0.915±0.004	0.958±0.002	0.6±0.1

**Table 13:** Estimated entropy and divergence of residual score distributions (our model), computed from per-frame scores $s_{t}$ at $f_{6}$ using the exact pipeline in this paper. Larger JSD indicates better separability between normal and anomalous residuals. Values are in nats.

Dataset	$H(P_{\mathrm{bg}})$	$H(P_{\mathrm{anom}})$	$\mathrm{JSD}(P_{\mathrm{bg}}\|P_{\mathrm{anom}})$
XD-Violence	2.60	2.95	0.08
Hockey Fight	2.10	2.45	0.12
UCSD Ped2	1.05	1.15	0.06

**Table 14:** Comparison of Average Precision (AP) on the XD-Violence dataset.

Model	Method	AP (%)
MGAFlow [20]	Unsupervised	75.3
DiffusionAD [32]	Unsupervised	68.0
STPM [33]	Unsupervised	61.0
MemAE [7]	Unsupervised	63.0
Flashback [34]	Zero-shot	75.1
Our model	Unsupervised	80.5

**Table 15:** Comparison of AUC and F1-score on the Hockey Fight dataset.

Model	Method	AUC	F1-Score
AnoGAN [8]	Unsupervised	0.63	0.57
GANomaly [35]	Semi-supervised	0.71	0.59
MemAE [7]	Unsupervised	0.73	0.65
CFA-HLGAtt [14]	Unsupervised	0.85	0.82
RTFM [21]	Weakly-supervised	0.85	0.73
ConvLSTM-AE [6]	Unsupervised	0.89	0.86
Our model	Unsupervised	0.92	0.85

**Table 16:** Comparison of AUC on the UCSD Ped2 dataset.

Model	Method	AUC
MPPCA [38]	Unsupervised	0.69
MDT [15]	Unsupervised	0.83
Conv2D-AE [23]	Unsupervised	0.85
ConvLSTM-AE [6]	Unsupervised	0.88
Conv3D-AE [39]	Unsupervised	0.91
STADNet [40]	Unsupervised	0.95
CR-AE [36]	Unsupervised	0.96
Optical Flow + STC + GAN [37]	Unsupervised	0.97
Multi-level Memory-augmented AMC [22]	Unsupervised	0.99
Our model	Unsupervised	0.96

**Table 17:** Compact comparison with reviewer-referenced baselines (conceptual).

Method	Mod.	Train	Key Idea
VAE-based UVAD (pose-driven) [41]	RGB/Pose	VAE	VAE reconstruction; uncertainty modeling.
RVAD-IGN (idempotent)	RGB	AE/Gen	Idempotent constraint (G(G(x))≈G(x)).
Attention–AE [14]	RGB	AE	Hierarchical local–global attention.
Ours	RGB+Flow	GAN	Early fusion; GRU–Attn; D feature matching.

**Table 18:** Effect of adding $L_{\mathrm{idemp}}$ (all else unchanged).

Setting	XD-V AP (%)	Hockey AUC	Ped2 AUC
$L_{\mathrm{DAS}}$ (ours)	80.5±0.2	0.920±0.003	0.960±0.001
$+\;L_{\mathrm{idemp}}$ ($\lambda_{\mathrm{id}}\;=\;0.05$)	80.7±0.2	0.923±0.003	0.961±0.002

## Data Availability

Data are contained within the article.
